# Long-Term Effectiveness of Intradiscal Culture-Expanded Mesenchymal Stem Cells (MSCs) with Platelet Products for Discogenic Low Back Pain

**DOI:** 10.3390/biomedicines13102365

**Published:** 2025-09-26

**Authors:** Nicholas Hooper, Joseph Ierulli, Chase Demarest, John Pitts, Oluseun A. Olufade, Christopher Williams

**Affiliations:** 1 Department of Rehabilitation Medicine, Emory University School of Medicine, 1364 Clifton Road NE, Atlanta, GA 30322, USA; 2 Regenexx Cayman, George Town, Grand Cayman KY1-1208, Cayman Islands; 3 Centeno-Schultz Clinic, 403 Summit Blvd 201, Broomfield, CO 80021, USA; 4 Department of Orthopedics, Emory University, 21 Ortho Lane, Atlanta, GA 30329, USA; 5 Interventional Orthopedics of Atlanta, Atlanta, GA 30329, USA

**Keywords:** low back pain, orthobiologics, intradiscal therapy, pain medicine, stem cells, mesenchymal stem cells, stromal cells, platelet-rich plasma, intradiscal, regenerative medicine

## Abstract

**Background/Objectives**: Low back pain (LBP) remains one of the leading causes of disability globally and contributes significantly to healthcare expenditures. Discogenic LBP, a subtype stemming from intervertebral disc degeneration, often provesrefractory to conventional treatment modalities. Regenerative orthobiologic therapies, including platelet-rich plasma (PRP), platelet lysate (PL), and mesenchymal stem cells (MSCs), have emerged as promising alternatives, though long-term outcomes and safety profiles are not yet well understood. **Methods**: This case series reports 13 patients treated between 2015 and 2016 at an outpatient interventional pain center who received intradiscal culture-expanded MSC injections with or without additional injections to other surrounding vertebral structures. There was no control group. Inclusion required patients to have discogenic LBP with or without radiculopathy and at least six years of completed follow-up data. Outcomes were assessed using Numeric Rating Scale (NRS), Functional Rating Index (FRI), and modified Single Assessment Numeric Evaluation (SANE) scores at multiple time points up to 10 years post treatment. **Results**: Thirteen patients met the inclusion criteria. Significant reductions in NRS and FRI scores were observed at 6 months, 3 years, and 6 years (*p* < 0.01). At 6 years, the average NRS score decreased by 2.50 points, FRI by 24.14 points, and SANE showed a 60% improvement. At 10 years, among the seven patients who responded, average SANE improvement was 78.1%. No adverse events were reported. **Conclusions**: This study presents the longest known follow-up data for intradiscal MSC therapy for discogenic LBP, demonstrating sustained improvements in pain and function. These findings support further investigation into combination orthobiologic therapies as a viable long-term treatment option for chronic LBP.

## 1. Introduction

Low back pain (LBP) is one of the leading causes of disability worldwide [1]. In the United States alone, it remains the third largest healthcare expenditure behind diabetes and ischemic heart disease, with reported costs exceeding USD 100 billion annually [2,3]. Overall lifetime prevalence of LBP is reported to be 80% or higher [3,4,5]. Common causes of low back pain include spondylosis, facet-mediated pain, discogenic pain, sacroiliac joint dysfunction, and myofascial pain [6,7]. Discogenic back pain is a subcategory of low back pain that is marked by pain related to the intervertebral disc [4,5,6,7]. The mechanisms surrounding discogenic pain are multifactorial but stem from degenerative changes to the disc which lead to structural changes, biochemical instability, localized inflammation, and nerve ingrowth of nociceptive unmyelinated nerve fibers [4,5,6,7].

Treatments for LBP can include pharmacological management, non-pharmacological approaches, and surgical approaches depending on the etiology [8,9,10]. Pharmacologic management of discogenic pain typically includes the use of nonsteroidal anti-inflammatory drugs (NSAIDs) and acetaminophen. Other adjunctive medications like gabapentin, muscle relaxants, and anti-depressants are commonly used to manage LBP patients [5,8,9,10]. However, many patients continue to suffer from LBP refractory to conservative care with medication, cognitive behavioral therapy, and physical therapy. These patients may then undergo minimally invasive therapy with injection-based therapy with corticosteroids and/or anesthetics to target a limited number of structures, or other interventional procedures like nerve ablation [5,9,10,11,12,13,14]. Despite a multitude of treatment options, studies have shown over 68% of patients with discogenic low back pain report chronic symptoms despite undergoing non-surgical treatments [5,15].

With the lack of consistent long-term success of many conventional therapies, there is greater interest in alternative interventions to provide ameliorative treatment. One therapy of interest is regenerative medicine involving orthobiologic treatment options. Orthobiologics are defined as substances that may be delivered via injection intended to repair or strengthen weakened musculoskeletal structures, and can include growth factors, cells, or proliferative solutions [16,17]. These therapies can include biologics like platelet-rich plasma (PRP) as well as cellular therapy with isolated or concentrated MSCs (i.e., mesenchymal stem cells, sometimes referred to as medicinal signaling cells or stromal cells) [16,17]. Several small clinical trials have evaluated the efficacy of regenerative therapies for discogenic LBP. Pettine et al. found that, of 26 patients suffering from degenerative disc disease who were treated with intradiscal autologous non-culture-expanded BM-MSCs, the average pain score significantly decreased up to 3 years and only 6 out of the 26 progressed to requiring surgical intervention [18]. Centeno et al. published a small case series of five patients treated with intradiscal autologous MSCs with a similar protocol to this study, with results at 4–6 yrs showing four of five patients had resolution of their disc protrusion, significantly reduced pain, improved function, and overall improvement [19]. Centeno et al. also published results of 33 patients treated with a similar protocol showing an average improvement of 60% over 6 years, and 85% had a reduction in disc bulge size [20]. In addition, Pang et al. reported that their two patients treated with umbilical cord MSCs had greatly improved functional and pain scores [21]. A recent systematic review in 2022 examining the use of intradiscal orthobiologics found that, of five studies that included the aggregate success rate at 6 months, the average value was 53.5% [22]. However, the study did not examine outcomes beyond 6 months. Likewise, multiple studies have demonstrated the efficacy of intradiscal PRP for the management of LBP [23,24,25]. Tuakli-Wosornu et al. found that patients who received intradiscal PRP had a significant improvement in both NRS and FRI when compared to a control group [23]. Overall, there have been six studies published to date using intradiscal autologous or allogeneic cultured cells, with no studies reporting on outcomes beyond 6 years [19,20,26,27,28,29].

The emergence of orthobiologic therapies as a treatment modality for axial back pain has allowed interventional pain management physicians to consider and treat the entire osteoligamentous and disc complex when managing musculoskeletal conditions, shifting from the long-standing model of treating a single primary pain generator [30,31,32,33]. However, there have been few studies examining the use of combination regenerative therapy, including PRP, platelet lysate (PL), and culture-expanded MSCs, in the management of LBP [34,35,36]. Here we present a case series reporting on the long-term pain and functional outcomes of patients with low back pain treated with a combination of intradiscal culture-expanded bone marrow-derived MSCs (BM-MSCs) with PL and/or PRP, in addition to other tissue targets within the osteoligamentous complex.

## 2. Materials and Methods

### 2.1. Patient Recruitment

Patients undergoing treatment for discogenic low back pain at an outpatient-based private practice interventional pain center were considered for inclusion in this study. A clinical registry was used to prospectively collect patient outcome data. All patients signed a written consent prior to participation, and this study was approved by an Institutional Review Board (Protocol Number: HHG2014-01). Prior to treatment and post treatment, patients received electronic surveys, via ClinCapture (Clinovo Clinical Data Solutions, Sunnyvale, CA, USA), to self-report outcomes and adverse events at intervals of one, three, six, twelve, eighteen, twenty-four months, and annually thereafter for up to twenty years. Up to five attempts would be made to contact participants electronically prior to patients being considered lost to follow-up. Any adverse events reported were reviewed and characterized by the treating physician and indexed.

Inclusion criteria for this study included the following: (1) patients with discogenic LBP with or without radiculopathy; (2) patients who were treated with intradiscal culture-expanded MSCs, platelet lysate (PL), and/or platelet-rich plasma (PRP); (3) patients enrolled in the registry with completed outcome data for a minimum of six years; (4) treatment completed between 1 January 2015 and 31 December 2016. Search criteria for the identification of patients with the registry included degenerative disc disease, axial low back pain, herniated disc, annular tear, disc herniation, and low back pain with or without radicular symptoms. Following the identification of patients meeting the inclusion criteria, a more extensive chart review was conducted to confirm the structures injected and the characteristics of the injectates utilized. Exclusion criteria for this study included the following: upon further chart review any patients that did not have a lumbar intradiscal injection and/or patients that received treatment outside of the predetermined treatment window.

A total of 23 patients were initially identified as receiving lumbar intradiscal injections, and based on the inclusion and exclusion screening criteria the number of patients used for the analysis was 13. Patient demographics were reviewed during the chart review and included the following data: gender, age, diagnosis, structures injected, and injectates.

### 2.2. Injectates

#### 2.2.1. Culture-Expanded Bone Marrow-Derived MSCs (BM-MSCs) and Platelet Lysate (PL)

A detailed description of the bone marrow aspiration, isolation, and expansion technique has been previously published [34,37,38]. In brief, a total of 90–120 mL bone marrow was aspirated from the posterior superior iliac spine under ultrasound or fluoroscopic guidance. Prior to the aspiration, it was recommended that patients abstain from the use of steroids for a minimum of six weeks prior and nonsteroidal anti-inflammatory medications for four to six weeks prior. For the aspiration, with the patient in the prone position and under sterile conditions, 20 mL of 1% lidocaine was used to anesthetize the skin and subcutaneous tissue around the posterior superior iliac spine (PSIS) on the left and right sides of the posterior pelvis. Following this, a sterile disposable trocar was used to manually enter the marrow cavity at 4–5 entry sites, aspirating 5 to 15 ccs per site, collecting a total of 45–60 cc of bone marrow on each side. The bone marrow aspirate was collected into heparinized syringes containing 1000 units of Heparin (Aurobinda pharma limited-NDC 63739-964-25) per mL of the syringe. The BMA was then transferred to a clean room laboratory for culturing. On the same day as the bone marrow aspiration, approximately 520 mL of venous blood was collected into a blood bag containing 80 ccs anticoagulant citrate dextrose solution A (ACD-A) and used to create platelet lysate (PL); yielding high concentrations of PDGFs. The PL was prepared by centrifuging the blood at 300× *g* for 23 min to separate platelet-rich plasma (PRP) from the red blood cells (RBCs). PRP was drawn off and stored at −20 °C overnight to produce pellet platelet bodies. Prior to utilization, the frozen PL was thawed in a water bath and then re-centrifuged at 1000× *g* for 6 min and the resulting supernatant was drawn off to produce the PL. Cell culture media was prepared using minimum essential medium (D-MEM or A-MEM) with 10% PL by volume, heparin at 2 IU/mL, and doxycycline at 5 μg/mL. Platelet lysate was also prepared from fresh whole blood to be used on the day of the injection utilizing the outlined steps above with the following modification. Instead of freezing the PRP overnight, after removal of the platelet poor plasma (PPP), the remaining platelet pellet was placed in a −80 °C freezer for 5–10 min and then the removed volume of PPP was used re-suspend the pellet and to create PL ready for immediate use.

For the culturing process, the BMA was centrifuged at 300× *g* for 6 min to the buffy coat from the RBC layer. A small aliquot of the buffy coat layer containing the nucleated cells was used to determine the nucleated cell count. Nucleated cells were seeded in complete culture medium and after 6–12 days in hypoxic culture (i.e., 5% oxygen) MSC colonies that developed were harvested by using an animal origin-free trypsin-like enzyme (TrypLE Select-Gibco, 12563). For the expansion of MSCs, cells were re-plated at a density of 4000–5000 cells/cm^2^ in complete culture medium (MEM + platelet lysate + heparin + doxycycline) and grown to approximately 85% confluence. Passage 0 consisted of primary cells derived from the nucleated cell population, and each subsequent subculture of MSCs was considered one further passage. After 2–5 passages, MSCs were harvested, washed, and suspended in PL with the addition of PRP (detailed below) in readiness for the injection.

#### 2.2.2. Platelet-Rich Plasma (PRP)

Patients underwent a venous blood draw of approximately 60 to 500 mL within 24 h of planned procedure. The anticoagulated whole blood was then centrifuged at 300× *g* to isolate PRP (red and white cell poor, high platelet concentration). PRP was then hard spun at 1000× *g* to produce platelet pellets, the platelet-poor supernatant (PPP) was removed, and an appropriate volume of PPP was re-added to re-suspend platelet pellets at the desired volume and concentration.

### 2.3. Injection Technique

Prior to performing the procedure, a small sample of the prepared MSCs was separated and used for determining cell count, sterility and endotoxin testing, and karyotyping. If abnormalities were discovered, the treating physician and patient were notified and the procedure was cancelled.

Under sterile conditions, all patients received percutaneous injections under multiplanar fluoroscopic guidance. Patients were positioned prone with a pillow under the abdomen to flatten the lumbar lordosis. The lumbar spine was cleaned with chlorhexidine and draped in sterile surgical fashion with the treating physician wearing a surgical scrub cap, and sterile gown and gloves. Using C-arm fluoroscopy, the anterior and posterior endplates of the desired disc level were aligned. The C-arm was obliqued so that the superior articular process of the lower level was in the middle third of the vertebral body. Then a 20-gauge introducer needle was advanced under intermittent fluoroscopy lateral to the SAP and posterior lateral to the disc. Then a 25-gauge needle was advanced through the introducer needle into the disc nucleus, confirming placement with anterior posterior and lateral fluoroscopic views. Then, 0.1 cc of Iodixanol (Visipaque™ NDC 0407-2223-06; GE Healthcare, Little Chalfont, UK) radiographic contrast was injected to confirm the nucleogram and then 1–2 cc of cultured expanded cells suspended in PL was injected. Patients received an intradiscal injection with or without a combination of injections into the following structures: zygapophyseal joints, transforaminal epidurals, multifidus muscles, supraspinous ligaments, interspinous ligaments, iliolumbar ligaments, sacroiliac joint, and overlying ligaments. Specific levels for the injections and laterality were determined by symptoms, physical examination, and imaging studies. The identified patients included in the analysis only received one injection during the study period. All patients were provided with post-procedure recovery instructions that provided a guideline for progressing physical activity for sixteen weeks following the date of treatment. See Table 1 for a description of the structures injected and the injectate details.

### 2.4. Outcomes

Treatment outcomes were measured by several patient-reported questionaries. Patients were asked to complete the Pain Numeric Rating Score (NRS) [39] and Functional Rating Index [40] at baseline and each follow-up time point. In addition, they were asked to complete a modified Single Assessment Numeric Evaluation (SANE) at each follow-up time point. The FRI is on a scale from 1 to 100, where 0 indicates no functional limitations and 100 represents severe disability [40]. The NRS is a pain scale ranging from 0 to 10, where 0 indicates no pain and 10 is the worst pain possible [39]. Lastly, the modified SANE asked patients to rate the percentage difference they felt following the treatment compared to pre-intervention [41]. This was reported as −100 (worsened) through +100 (improved); however, negative ratings were truncated as 0 in order to align with the standard SANE rating. Patients with incomplete outcome questionaries were contacted for a final attempt to obtain a 10-year SANE score to be included in the outcomes analysis.

### 2.5. Statistical Analysis

Continuous variables were described using the mean and standard deviation (SD). Individual differences between post-treatment time points and baseline were calculated for NPS and FRI metrics. Wilcoxon signed-rank tests were used to assess whether post-treatment scores differed significantly from baseline. Additionally, effect sizes were calculated for each time point versus baseline for NRS and FRI metrics. Hedges G was calculated due to the expected small sample size. All statistics were conducted using R Studio (version 2025.05.01) [42].

## 3. Results

During the data review process, there were a total of 23 patients that received intradiscal injections during the treatment window between 1 January 2015 and 31 December 2016. A total of 13 patients met our inclusion criteria. Of these 13 patients 3 underwent multiple procedures at least 2 years apart. The average age of patients undergoing treatment was 44.1 years, with the youngest patient being 18 years old and the oldest being 70 years of age (Table 1). Of those included, only 23.1% (*n* = 3) were female, while 76.9% (*n* = 10) were male. All of the patients underwent a combination of MSC injection with either PRP or PL into the lumbar intradiscal region. The average MSC count injected into the disc was 21.4 million (range of cells injected was 6.1–45 million).

Other frequently targeted areas included transforaminal ESI (42.9%), intraarticular facets (35.7%), and supraspinous interspinous, and iliolumbar ligaments (7.1%). Of the 16 procedures, no adverse events were reported.

**Figure 1:**
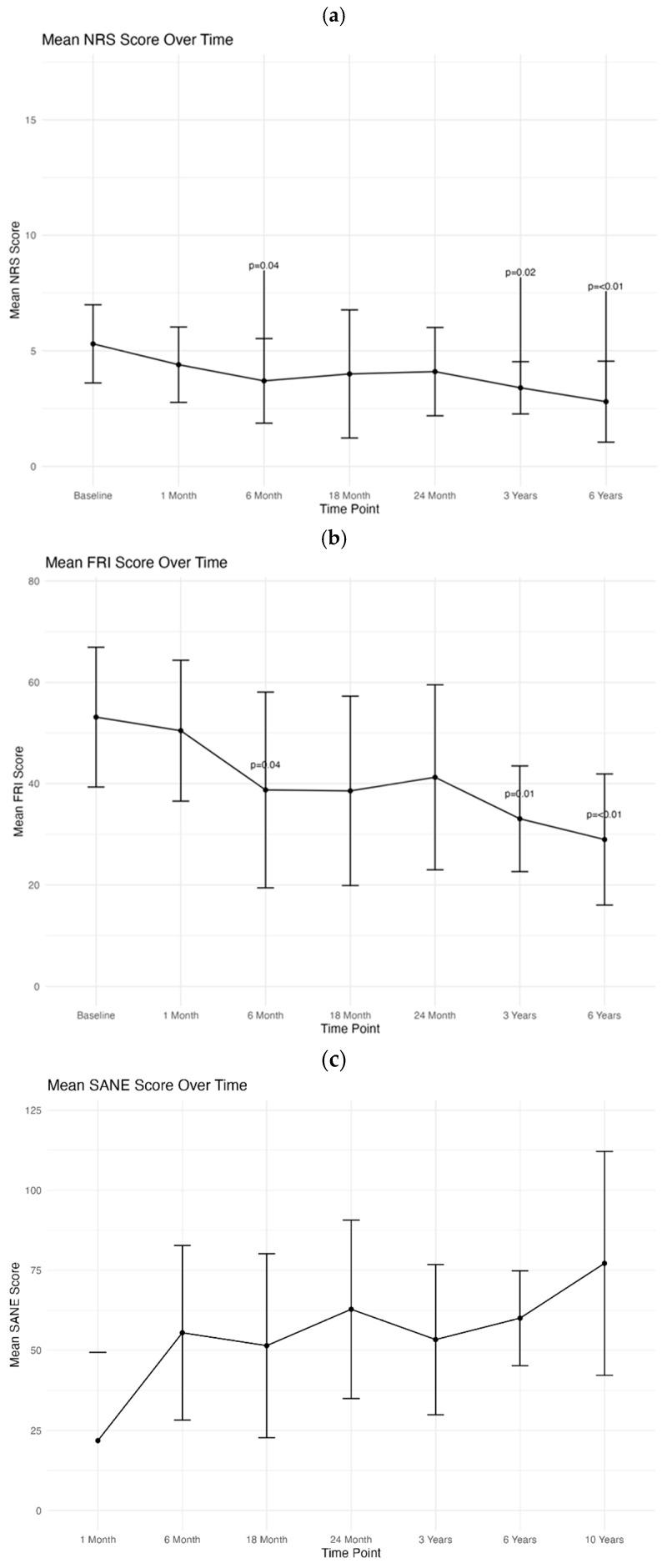
(**a**) Longitudinal trend of NRS means at baseline, 1 month, 6 months, 18 months, 2 years, 3 years, and 6 years. Bars represent 95% confidence intervals. Significant *p* values (i.e., *p* < 0.05) are depicted on the bar. (**b**) Longitudinal trend of FRI means at baseline, 1 month, 6 months, 18 months, 2 years, 3 years, and 6 years. Bars represent 95% confidence intervals. Significant *p* values (i.e., *p* < 0.05) are depicted on the bar. (**c**) Longitudinal trend of SANE means at 1 month, 6 months, 18 months, 2 years, 3 years, 6 years, and 10 years. Bars represent 95% confidence intervals.

A complete outcomes dataset that included the NRS, FRI, and SANE averages are reported in Table 2 and shown in Figure 1a–c. Significant improvement in NRS scores was seen at 6 months, 3 years, and 6 years when compared to baseline. At 6 years there was an overall 2.50-point decrease in NRS scores when compared to baseline (*p* = <0.01). Improvement in mean FRI scores reached statistical significance at 6 months, 3 years, and 6 years. At 6 years there was an overall 24.14-point drop in average FRI scores when compared to baseline (*p* = <0.01). The modified SANE scores showed an average improvement of 60% at 6 years. The peak improvement in SANE scores was noted at 2 years (62.8% improvement). Of the 16 procedures completed, only once did a patient report no improvement in their symptoms at 6 years. Outcomes were analyzed between patients receiving intradiscal only versus intradiscal with other structures injected as well (i.e., facet joints, epidurals, ligaments, sacroiliac joints, etc.), as shown in Table 3. Incomplete patient reported outcomes were also included at 10 years and are reported in Table 4.

Out of the 13 patients, 7 reported long-term outcomes at 10 years. Of these patients, all 7 responded to SANE outcome. Of these 7 patients, the average SANE was 78.14. Only 1 patient reported no improvement. Half of the patients reported a SANE over 90. Only 3 out of the 8 patients reported NRS scores. All 3 of the patients reported an NRS score of 1.

## 4. Discussion

In the present study, we report the longest reported outcomes to date for an intradiscal treatment with autologous culture-expanded mesenchymal stem cells. In addition to safety, we aimed to determine the long-term efficacy of intradiscal BM-MSCs for the treatment of low back pain. In a case series of 13 patients from a patient registry, we have observed sustained improvement in pain and functional outcomes up to 10 years.

This case series supports the use of multifactorial regenerative medicine injections performed under image guidance for the effective treatment of discogenic low back pain. Of the 13 patients, all underwent image-guided treatment with intradiscal culture-expanded MSCs along with a combination of platelet lysate (PL) and/or platelet-rich plasma (PRP). Significant improvements were observed in both functional outcomes and pain scores at 6 months, 3 years, and 6 years. Notably at the 6-year follow-up, a 2.50-point reduction in NRS scores, a 24.14-point decrease in FRI scores, and a 60% improvement in SANE scores (*p* < 0.01) were observed. Of the 16 overall procedures only one showed no long-term symptom improvement at 6 years. Additionally, of the seven patients reporting 10-year outcomes, the average SANE was 78%. Given the lack of 10-year outcomes, only the SANE average was reported, with individual values reported in Table 4, thus demonstrating continued improvement from our 6-year follow-up time point.

As stated above, there were no adverse events reported in this case series of patients. In order to determine this, patients were asked about increased pain and signs of symptoms of infection at each time point. No cases of discitis or complications were reported at the interventional pain center during the 2015–2016 treatment window included in this study. Given no reported adverse events, no patients underwent further monitoring with MRI or tissue sampling. However, in an effort to gain a better understanding of the overall prevalence and incidence of complications related to intradiscal injection, a deeper analysis of all intradiscal patients treated at a single outpatient clinical site from its inception until the time of the submission of this publication was conducted. There were a total of 127 patients that received intradiscal injections. Of these 127 patients, 26 received repeat treatments at a later date, and 5 were diagnosed with suspected discitis (3.94%). Discitis was only suspected as none of the five patients underwent tissue sampling to confirm the diagnosis. Three out of the five cases (60%) occurred in patients who received at least one subsequent intradiscal injection. Given this, there may be an association or risk of repetitive intradiscal injections and an increased risk for the development of discitis. One potential reason for this is that lumbar discs have been shown to have a diverse microbiome and dysbiosis, thus injection could potentially lead to progressive degenerative disc disease or discitis, especially if there is an abundance of Propionibacterium acnes (P acnes) [43]. Additionally, many of the suspected cases of infectious discitis may actually be aseptic discitis. This assumption is within reason, given the suspected mechanism of action of biologics that includes a transient pro-inflammatory state during the activation of the healing cascade. No additional adverse events or complications have been identified to date, including the need for surgical intervention.

The use of intradiscal PRP and MSCs for the treatment of discogenic low back pain, though sparse, is supported in the literature. In one study, 29 patients with discogenic LBP who failed conservative management were treated with intradiscal PRP injections. The results were similar to our findings, with a 2.6-point decrease in average pain scores using NRS, as well as functional scores improving by approximately 20% at one year post treatment [44]. In another study, 37 patients who received intradiscal injections of high-concentration PRP were compared to 29 patients who received the standard concentration of PRP [45]. FRI and NASS outcomes showed significant improvements of 3.4 and 46.4, respectively, in the high concentration group at an average of 18 months post procedure [22]. A 2022 systematic review examining the use of intradiscal orthobiologic injections found of 37 total studies, only 5 examined the use of MSCs. The review suggested that intradiscal biologic agents were effective treatment options for discogenic LBP, with >50% of patients showing >50% relief at 6 months [22]. The review did not examine any long-term outcomes further than 6 months. Additionally, only one of these five studies examined the combination of culture-expanded MSCs and PRP. Like our study, the five studies also analyzed the effectiveness of biologic agents in the treatment of low back pain, showing similar good outcomes. Where they are markedly different is in our use of combination regenerative therapy and examination of long-term outcomes, underscoring a gap in the literature that warrants further study.

A notable limitation of this study is its smaller sample size. After inclusion criteria were accounted for, a total of 13 patients were included for data analysis. Related to the relatively small sample size is the demographic makeup. The patient population was predominantly male (78.6%) with an average age of 44.1 years. Considering that the incidence of LBP increases with age, as does comorbid conditions, this sample may not accurately reflect the broader population of patients with discogenic low back pain. Given both the small sample size and demographic make-up, generalizability may be severely limited. As such, the conclusions of this study may not be generalizable to the greater population. An additional limitation of the data is the level of missingness. Due to this, only the average of the 10-year SANE scores was reported. Efforts were made to reduce the number of missing outcomes by sending multiple reminders to patients to complete the surveys at each time point. However, it is possible that non-response bias may have played a role. Another limitation of the study design is the lack of a control group. Without a comparison group, improvements cannot be solely attributed to the intervention. It is possible that natural disease course and placebo effect may have contributed to the benefits observed. An additional limitation is that most of the patients treated did receive other structures injected at the same time of the intradiscal treatment. This significantly limits the interpretation of the results to the intradiscal treatment alone. Additional structures treated were identified based on the correlation of the patient’s symptomatic history, physical examination findings, and imaging results. The decision was made to not treat the lumbar discs in isolation, with the understanding that there is an intricate interplay and complexity to degeneration in the spine and the involvement of the functional spinal unit as it relates to spinal pathology and presenting symptoms. Analyzing the outcomes between the patients that had intradiscal injections only versus other structures in addition to the intradiscal injection failed to show significant differences in any of the outcome measures as well. Lastly, the lack of standardization of PRP and MSC treatment also provides another barrier in limitations as the specific concentrations and formulations of PRP, PL, and MSCs varied among patients, making it difficult to assess the exact efficacy of each component or compare outcomes to those from other studies using standardized protocols. In addition, some patients also underwent more than one treatment. A barrier for the utilization of orthobiologics, such as in this study, is the high cost associated with its preparation and administration, which is not typically covered by insurance companies. This financial burden may limit accessibility for many patients and hinder broader applications of regenerative injection techniques in clinical practice.

## 5. Conclusions

Our study examines the long-term safety and efficacy of orthobiologics as an alternative treatment for discogenic low back pain. Treatment with autologous MSCs, PRP, and PL effectively demonstrates improvement in functional and pain metrics with no serious adverse events occurring during the 10-year follow-up period. Although these data in conjunction with other recent studies are encouraging, research continues to be hindered by small study sizes and methodological heterogeneity, which highlights the limitations of this study and the continued need for additional research. As such, more comprehensive, large-scale randomized controlled trials (RCTs) are necessary to further validate these findings. However, the promising positive outcomes of this dataset do suggest the efficacy of this regenerative medicine approach for discogenic pain and further provide a methodological foundation by which larger-scale RCTs can be developed. This study reinforces the importance of continued research into regenerative medicine as a potential treatment approach for low back pain as it may allow more patients access to safer and more effective care.

## Figures and Tables

**Table 1:** Patient demographics, diagnosis, and intervention performed.

Demographics			Diagnosis	Procedure		
Patient	Age	Gender		Injectate	Intradiscal MSC Dose (Millions)	Additional Structures Injected
1	44	M	L5-S1 DDD, radiculopathy	MSCs, PRP	20.0	L5-S1 intradiscal L5/S1 transforaminal and L4-S1 supraspinous and interspinous ligaments
2	44	M	L4-S1 DDD, radiculopathy	MSCs, PL,	21.0	L4-L5 and L5-S1 intradiscal bilateral L4-S1 intraarticular facet Right L4-L5 and L5-S1 epidural with PL
3	33	M	L5-S1 discogenic pain with annular tear	MSCs, PL, PRP	10.0	L5-S1 intradiscal Bilateral L2-L3, L3-L4, L4-L5, and L5-S1 epidural with PL
4	68	M	Multilevel DDD	MSC, PRP	15.4	L4-L5 intradiscal
5	40	M	L4-L5 and L5-S1 herniated disc with annular tear, radiculopathy	MSC, PRP, PL	25.1	L4-L5 and L5-S1 intradiscal Bilateral L4-L5 and L5-S1 intra-articular facet Right L4-L5 and L5-S1 TFESI with PL
6	27	M	L5-S1 disc herniation, radiculopathy	MSC, PL, PRP	30.0	L4-L5, L5-S1 intradiscal
7	70	F	L3-L4 and L5-S1 disc protrusion, DDD	MSC, PL, PRP	45.0	L5-S1 intradiscal Left L4-L5 and L5-S1 TFESI Left L3-L4 and L4-L5 facet
8	62	M	L2-L3 herniated disc, radiculopathy	MSC and PL	10.0	L2-L3 intradiscal
9	20	F	L5-S1 herniated disc, radiculopathy	MSC, PL, SCP	9.0	L5-S1 intradiscal
10	52	M	DDD, L5-S1 annular tear	MSC, PL, PRP	6.1	L5-S1 intradiscal
11	18	M	L5-S1 herniated disc	MSC, PL	27.0	L5-S1 intradiscal
12	67	F	L4-L5 and L5-S1 disc herniation with central canal stenosis, annular tear	MSC, PL	33.1	L4-L5 intradiscal
13	46	M	Herniated disc with disc building at L2-L3, L3-L4, L4-L5, and L5-S1 radiculopathy	MSC, PL, PRP	27.1	L3-L4, L4-L5, and L5-S1 intradiscal Right L3-L4, L4-L5, L5-S1 TFESI Bilateral L4-L5 and L5-S1 facet injections

**Table 2:** Outcome means at each time point.

Outcome	Mean (95% CI)	SD	N	*p*-Value	Hedges G
NRS					
Baseline	5.30 (4.45, 6.15)	1.69	16	NA	NA
1 Month	4.40 (3.31, 5.49)	1.63	10	0.11	0.55
6 Months	3.70 (2.56, 4.81)	1.83	11	**0.04**	0.88
18 Months	4.00 (1.60, 6.40)	2.77	7	0.34	0.39
24 Months	4.10 (2.85, 5.35)	1.91	10	0.07	0.67
3 Years	3.40 (2.66, 4.14)	1.13	9	**0.02**	1.17
6 Years	2.80 (1.92, 3.68)	1.75	12	**<0.01**	1.14
FRI					
Baseline	53.13 (45.70, 60.56)	13.80	16	NA	NA
1 Month	50.45 (41.81, 59.09)	13.91	11	0.21	0.20
6 Months	38.75 (27.40, 50.10)	19.32	12	**0.04**	0.85
18 Months	38.57 (20.36, 56.78)	18.70	7	0.34	0.76
24 Months	41.25 (27.61, 54.89)	18.27	10	0.21	0.70
3 Years	33.06 (25.58, 40.54)	10.44	9	**0.01**	1.52
6 Years	28.96 (21.38, 36.54)	12.95	12	**<0.01**	1.56
SANE					
1 Month	21.82 (5.00, 38.64)	27.50	11	NA	NA
6 Months	55.45 (39.00, 71.90)	27.25	11	0.06	NA
18 Months	51.43 (25.44, 77.42)	28.68	7	**0.04**	NA
24 Months	62.78 (44.18. 81.38)	27.85	9	**0.04**	NA
3 Years	53.33 (37.32, 69.34)	23.45	9	**0.02**	NA
6 Years	60.00 (50.70, 69.30)	14.83	11	**0.01**	NA

Table 2 depicts each outcome at baseline, 1, 6, 18 months, 2, 3, and 6 years. Wilcoxon signed-rank tests were used to assess whether post-treatment scores differed significantly from baseline. Significance was defined as a *p*-value of less than 0.05. Significant *p*-values are in bold. Hedges G was calculated for each time point versus baseline. A value of 0.2 was considered a small effect, 0.5 a medium effect, and >0.8 a large effect.

**Table 3:** Outcomes stratified by patients receiving only intradiscal procedures versus procedures involving additional structures.

Outcome	Mean (SD)	Mean (SD)	*p*-Value
	Intradiscal Only	Intradiscal With Associated Structures	
NRS			
Baseline	5.57 (2.15)	5.10 (1.37)	NA
1 Month	5.25 (2.15)	3.86 (1.86)	0.10
6 Months	2.33 (1.15)	4.00 (1.69)	0.10
18 Months	2.50 (0.71)	4.60 (3.13)	0.22
24 Months	4.67 (2.52)	4.29 (2.21)	0.65
3 Years	3.75 (0.96)	3.29 (1.11)	0.49
6 Years	2.00 (1.83)	2.83 (1.75)	NA
FRI			
Baseline	57.92 (13.17)	50.25 (14.02)	NA
1 Month	46.88 (6.57)	52.50 (16.69)	0.46
6 Months	20.83 (8.04)	41.00 (18.40)	**0.01**
18 Months	35.00 (NA)	40.00 (20.71)	NA
24 Months	36.67 (12.33)	43.57 (20.54)	0.56
3 Years	30.62 (10.87)	32.14 (9.98)	0.57
6 Years	22.50 (12.08)	28.96 (12.94)	NA
SANE			
1 Month	12.50 (15.00)	27.14 (32.51)	NA
6 Months	80.00 (17.32)	48.59 (22.93)	**0.05**
18 Months	67.50 (24.75)	45.00 (30.00)	0.40
24 Months	48.33 (43.68)	60.00 (30.69)	0.48
3 Years	45.00 (20.82)	58.57 (24.12)	0.36
6 Years	67.50 (15.00)	60.00 (14.83)	NA

**Table 4:** Depicts individual patient 10-year SANE outcomes.

Patient	10-Year SANE (%)
1	95
4	90
7	85
8	100
11	0
12	95
13	75

## Data Availability

De-identified data available upon reasonable request.

## Abbreviations

The following abbreviations are used in this manuscript:

NRS	Numeric Rating Scale
FRI	Functional Rating Index
SANE	Single Assessment Numeric Evaluation
PRP	Platelet-rich plasma
PL	Platelet lysate
MSC	Mesenchymal stem cell
LBP	Low back pain
